# Routine childhood vaccination among ethnocultural groups in Canada during the COVID-19 pandemic: A national cross-sectional study

**DOI:** 10.1016/j.pmedr.2023.102435

**Published:** 2023-09-25

**Authors:** Robin M. Humble, Eve Dubé, Joanne Olson, Shannon D. Scott, Shannon E. MacDonald

**Affiliations:** a Faculty of Nursing, University of Alberta, Edmonton, Alberta, Canada; b Department of Anthropology, Laval University, Quebec City, Quebec, Canada

**Keywords:** Routine, Vaccine, Child, Racialized minority, COVID-19

## Abstract

Some ethnocultural groups in Canada experience low routine childhood vaccination, with social locations and discriminations contributing to inequities. This study aimed to characterize routine childhood vaccination in the context of the COVID-19 pandemic, including the influence of discriminatory experiences when accessing health services. We conducted a cross-sectional national survey to assess parents’ acceptance of routine vaccines for their children ≤ 17 years in Oct/Nov 2021. Descriptive statistics were used to explore differences among ethnocultural groups and logistic regression to assess associations with parents’ low acceptance. Of 2531 parents, 21.8 % self-identified as Racialized minorities, 7.7 % Indigenous, 23.3 % newcomers, 10.0 % spoke minority languages most often, and 69.6 % belonged to a reference group who did not report these characteristics. Statistically significant findings included 36.6 % of Indigenous parents reporting that the pandemic made them realize that routine vaccines were more important compared to 16.7 % of newcomers. Discrimination/racism when accessing health services was most often experienced by Indigenous (27.8 %) and Racialized minorities (20.2 %), compared to the reference group (4.8 %). Racialized minorities were more likely to report low acceptance of routine vaccination (aOR = 2.19, 95 % CI: 1.18–4.05), and younger parents and those with only preschool-aged children were less likely to have low acceptance (aOR = 0.59, 95 % CI: 0.37–0.94; aOR = 0.53, 95 % CI: 0.36, 0.79). Low acceptance was associated with everyday stress preventing vaccination (aOR = 2.18, 95 % CI: 1.41–3.38). Public health decision-makers should ensure equitable access to routine childhood vaccination that targets the inclusion of ethnocultural groups, who may experience disproportionate barriers and low acceptance.

## Highlights

• Discrimination/racism when accessing healthcare was most often experienced by Indigenous and Racialized minorities.
• Racialized minorities were most likely to report low acceptance of routine childhood vaccines during the COVID-19 pandemic.
• Parents’ low acceptance was associated with everyday stress preventing access to childhood vaccines during the pandemic.
• Vaccination programs should target ethnocultural groups who may experience disproportionate barriers and low acceptance.

## 1 Introduction

An ethnocultural group is defined by shared characteristics unique to that group, such as country of origin, language, self-identified ethnicity, cultural traditions, or physical traits (Government of Canada, 2005). Canada is an ethnoculturally diverse nation of 37 million people, of which 6.2 % self-identify as Indigenous (i.e., First Nations, Métis, and Inuit) (Government of Canada. Statistics Canada., 2019), 21.9 % are newcomers (i.e., born outside of Canada), 26.5 % self-identify as a Racialized minority (i.e., persons who are non-Caucasian in race or non-white in colour) (Government of Canada. Statistics Canada, 2023), and 12.7 % primarily speak a minority language (Government of Canada. Immigration, Refugees and Citizenship Canada., 2021, Government of Canada. Statistics Canada., 2022). Intersectionality is the interconnection of social determinants and, as a theoretical approach, acknowledges overlapping and interdependent systems of discrimination (Wesp et al., 2018). Intersectional social determinants may include ethnocultural identities (e.g., newcomers, Racialized minority, Indigenous) and social locations (e.g., household income, education) that, when combined, may perpetuate health inequities (Kholina et al., 2022 Dec, Luxenburg et al., 2023 Jan).

Historically, routine childhood vaccination in Canada has eradicated polio and caused a 90–95 % reduction in pertussis and measles infections (Dummer et al., 2012 Sep). Even so, in 2019, only 78 % of 2-year-old children in Canada had received all recommended doses of pertussis-containing vaccine (Government of Canada. Public Health Agency of Canada., 2022). Measles vaccination coverage for children at 7 years of age within Canada remains at 83.3 % (Government of Canada. Public Health Agency of Canada., 2022), 10 % below national targets (Government of Canada, 2020), contributing to periodic outbreaks (Rudnick et al., 2022 Jan 31). Although a wealthy country with universal healthcare (e.g., free health services), low vaccination coverage and increased incidence of vaccine-preventable diseases has been noted within some ethnocultural groups (i.e., Racialized minority, Indigenous, newcomers) (Rudnick et al., 2022 Jan 31, Carpiano and Bettinger, 2016 Dec, MacDonald et al., 2022, Stangl et al., 2019). Vaccination inequities are often attributed to differential access to health services, such as a delay in healthcare coverage, language barriers, a lack of culturally relevant care, disproportionate constraints (Zghal et al., 2021 Sep, Pandey et al., 2022 Feb, MacDonald et al., 2022), and experiences of discrimination when accessing health services (Lin, 2022 Aug, Government of Canada. Public Health Agency of Canada., 2021).

During the first waves of the COVID-19 pandemic (i.e., 2020 and 2021), a decline in routine childhood vaccination was reported by many countries (Abu-rish et al., 2022, McDonald et al., 2020, Shapiro et al., 2022). However, few Canadian studies (Lin, 2022 Aug, Dong et al., 2022, Lee et al., 2022) have explored acceptance of routine childhood vaccination among ethnocultural groups during the COVID-19 pandemic. Furthermore, it is not well understood if COVID-19 infection prevention measures (i.e., stay at home orders, school closures) created further challenges for some parents of ethnocultural groups to access routine vaccines for their children during the pandemic.

Therefore, this study aimed to characterize perceptions and acceptance of routine childhood vaccination during the COVID-19 pandemic, among a national sample of parents who self-identified as Indigenous, Racialized minorities, newcomers, those who primarily speak a minority language, and a reference group of parents who did not identify with any of these ethnocultural characteristics. This study sought to provide information for public health decision-makers that will support inclusive and equitable access to routine childhood vaccination for ethnocultural groups within Canada, who may experience disproportionate barriers to services.

## 2 Methods

### 2.1 Study design and participants

We conducted a cross-sectional national online survey in Oct/Nov 2021, just before the first pediatric COVID-19 vaccine (BNT162b2, Pfizer-BioNTech, 10 µg/dose) was approved for children aged 5–11 years in Canada. Survey respondents were randomly selected from a panel of > 400,000 adults from a well-established national polling firm, who lived in Canada, were proficient in reading French or English, and who had access to the internet (Leger Marketing Inc. Leger Opinion LEO Panel [Internet]., 2023). An invitation to participate and informed consent was sent by email. The overall survey sample (N = 6,026) was representative for population size in all provinces, and by age and sex, based on the latest Census data (Government of Canada, 2016). To ensure rigor and validity (Eysenbach, 2004 Sep 29), embedded consistency questions were cross referenced for respondent authenticity and battery questions monitored for inattentiveness and straight lining. Respondents had unique URLs and 15 % were contacted by telephone for identity verification. Supplementary Table A1 provides the quality Checklist for Reporting Results of Internet E-Surveys (Eysenbach, 2004 Sep 29).

We purposively sampled minimum quotas of targeted populations. This included respondents who were primary caregivers to one or more children ≤ 17 years old in their home, respondents who self-identified as Indigenous (i.e., First Nations, Métis, and Inuit), Racialized minorities, those who spoke minority languages most often, and newcomers. We estimated the minimal sample size of the target population groups to be 402, based on the maximum variability possible in the outcome variable in the population (i.e., a proportion of 0.50), with a margin of error of +/- 5 % and 95 % confidence intervals (CIs).

The 75-question survey took approximately 19 min to complete. The survey instrument was developed by drawing from previously validated questions about perceptions of routine vaccination (i.e., 5Cs psychological antecedents of vaccination) (Betsch et al., 2018), a previous survey of Canadian parents’ acceptance of routine childhood vaccination (Dubé et al., 2018), areas of focus for our policy partners (including the National Advisory Committee on Immunization Secretariat), and expertise of our national team of immunization researchers. The draft survey was reviewed by public health experts, pre-tested with team members, and pilot tested with members of the public and revised accordingly. This study received approval from the Health Research Ethics Board at the University of Alberta.

### 2.2 Measures

Our outcome variable was parents’ self-reported acceptance of routine vaccines for their children during the COVID-19 pandemic. Respondents were asked, “If your child was due to receive a routine vaccine (e.g., MMR/ measles, whooping cough, rotavirus) during the pandemic (since March 2020) did you, or would you, have them get it?” with the following response options: 1) Yes, my child was due for a routine vaccine and they received it, or I would have them get it if one was due; 2) No, they did not receive it, or I would not have them get it if one was due; and 3) I don’t know. For binary analysis, ‘no’ and ‘I don’t know’ categories were combined and are defined as low vaccination acceptance.

Predictor variables were based on determinants of routine vaccination including: the 5Cs psychological antecedents of vaccination (Betsch et al., 2018), influenza vaccination behaviors, mandated routine and COVID-19 vaccination, experiences of discrimination and/or racism when accessing health services, and how the pandemic has changed parents’ perceptions about routine vaccination. Ethnocultural variables included: self-identified Indigenous or Racialized minority, newcomer (i.e., born outside of Canada), spoke minority languages most often at home, and an intersectional variable of mutually exclusive ethnocultural categories (i.e., one ethnocultural identity of Indigenous, Racialized minority, newcomer, or language minority; two identities of newcomer, Racialized, and/or language minority; three identities of newcomer, Racialized, and language minority; and a reference group of parents who did not self-identify with any of these ethnocultural identities). Sociodemographic variables included: province, age, level of education, employment status, annual household income, gender, marital status, and number and ages of children. Survey questions are provided as supplementary Table A2.

### 2.3 Statistical analysis

We calculated descriptive statistics (i.e., frequencies and percentages) of the predictor variables, in addition to 95 % CIs to explore differences in parents’ vaccination perceptions among ethnocultural intersectional groups. We then assessed the association between parents’ low acceptance of routine childhood vaccination and the predictor variables using binary logistic regression. We reported both unadjusted and adjusted odds ratios with 95 % CIs. Variables included in the adjusted model were those previously associated in the literature with routine childhood vaccination, in addition to variables with a p-value below 0.20 in the unadjusted model. Multicollinearity was assessed between variables, and all had a variance inflation factor below 5. Due to the online survey completion requirements, no data were missing. SPSS version 26.0 (IBM, Chicago, IL, USA) was used for the descriptive and regression analyses.

## 3 Results

### 3.1 Characteristics of the sample

Of the 2531 parents in our sample, 21.8 % self-identified as Racialized minorities, 7.7 % Indigenous, 23.2 % were newcomers to Canada, 10.0 % spoke minority languages most often, and 69.6 % belonged to a reference group who did not report these ethnocultural characteristics (Table 1). Further characteristics are provided in supplementary Table A3.

**Table 1:** Sociodemographic characteristics of a sample of Canadian parents of children aged ≤ 17 years (N = 2531), October/November 2021.

**Characteristic**	**Category**	**Total** **n (%)**
**Ethnocultural characteristics**		
**Ethnic or cultural origin**	White	1769 (69.9)
	Racialized minority^1^	554 (21.8)
	Indigenous^2^	194 (7.7)
	Prefer not to answer	14 (0.6)
**Newcomer status**	Canadian born	1945 (76.8)
	Born outside of Canada	586 (23.2)
	Arrived 2016–2021 (n = 195)	
	Arrived before 2016 (n = 391)	
**Newcomer citizenship status (N = 586)**	Canadian citizen	346 (59.0)
	Permanent resident^3^	200 (34.1)
	Temporary resident^4^	37 (6.3)
	Refugee	1 (0.2)
	Prefer not to answer	2 (0.3)
**Language spoken most often at home**	English	1487 (58.8)
	French	790 (31.2)
	Minority languages^5^	254 (10.0)
**Intersectionality of sample**^6^ (mutually exclusive groups)	Indigenous	194 (7.7)
	Newcomer	138 (5.5)
	Racialized minority	119 (4.7)
	Language minority^7^	0 (0)
	2 Intersecting identities (newcomer, Racialized, or language minority)^8^	273 (10.8)
	3 Intersecting identities (newcomer, Racialized, and language minority)^8^	191 (7.5)
	Reference group	1616 (63.8)
**Socioeconomic characteristics**		
**Province of residence**	British Columbia	257 (10.2)
	Alberta	289 (11.4)
	Prairies^9^	163 (6.4)
	Ontario	789 (31.2)
	Quebec	876 (34.6)
	Atlantic provinces^10^	157 (6.2)
**Age**	15–29 years	278 (11.0)
	30–39 years	983 (38.8)
	40–49 years	873 (34.5)
	50–59 years	336 (13.3)
	≥60 years	61 (2.4)
**Highest level of education**	High school or less	297 (11.7)
	Non-university certificate or diploma(college/apprenticeship)	829 (32.8)
	University degree/bachelor’s or more than bachelor’s	1391 (55.0)
	Prefer not to answer	14 (0.6)
**Employment status**	Full-time (≥37 h per week)	1775 (70.1)
	Part-time (<37 h per week)	336 (13.3)
	Unemployed	250 (13.8)
	Prefer not to answer	70 (2.8)
**Annual household income**	<$40,000	271 (10.7)
	$40,000–79,000	646 (25.5)
	≥$80,000	1430 (56.5)
	Prefer not to answer	184 (7.3)
**Gender**	Woman	1533 (60.6)
	Man	991 (39.2)
	Gender minority^11^	7 (0.3)
**Marital status**	Married/common-law	2107 (83.2)
	Not married or common-law	408 (16.1)
	Prefer not to answer	16 (0.6)
**Number of children in household (0**–**17 years old)**	1 child	1228 (48.5)
	2 children	983 (38.8)
	3 or more children	320 (12.6)
**Age category of child(ren)**	Preschool-aged child(ren) only (0–6 years old)	878 (34.7)
	School-aged child(ren) only (7–17 years old)	1246 (49.2)
	Pre- and school-aged children (0–17 years old)	407 (16.1)

Notes.

1 Racialized minority groups including Black, Latin/Central American, Arabic/West Asian/North African, East Asian, South Asian, and any respondents who selected one of these groups and white.

2 Indigenous respondents are individuals who self-identified as First Nations, Métis, or Inuk.

3 Permanent resident refers to a landed immigrant.

4 Temporary residents include non-permanent residents such as those in Canada on a work or study visa.

5 Appendix Table A3 provides minority languages spoken most often at home.

6 Mutually exclusive categories of respondents who self-identify as Indigenous, belonging to a Racialized minority group, newcomers to Canada, those who spoke a minority language most often at home, and a reference group of parents who do not self-identify with any of these categories.

7 All respondents who spoke a minority language most often at home also self-identified as either a Racialized minority or newcomer.

8 Respondents who self-identified as Indigenous did not self-identify as a newcomer or a Racialized minority and spoke English or French most often at home.

9 Prairie provinces include Saskatchewan and Manitoba.

10 Atlantic provinces include PEI, Nova Scotia, New Brunswick, and Newfoundland and Labrador.

11 Respondents who selected one of the following categories: gender non-conforming, transgender, two-spirit, and “not listed please specify”.

### 3.2 Descriptive statistics

**Fig. 1:**
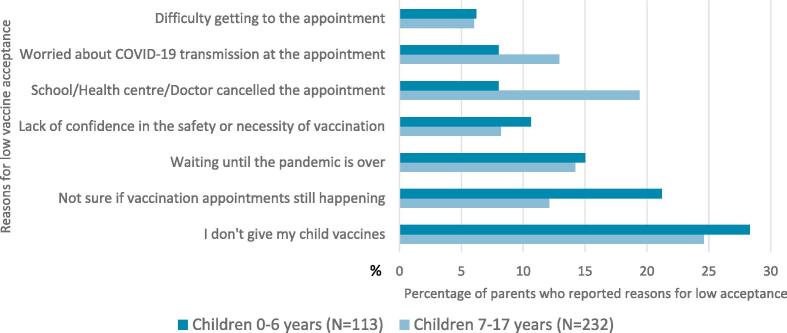
**A sample of Canadian parents’ reasons for low acceptance of routine vaccines during the pandemic for their children aged 0**–**6 years (n = 113) & 7**–**17 years (n = 232)** *Respondents provided reasons for low acceptance separately for children 0–6 and 7–17 years.

Eighty-seven percent (87.9 %) of parents reported that they would accept routine vaccination for their children during the COVID-19 pandemic, whereas 8.1 % had no intention and 4.0 % remained undecided (Table 2). Parents’ reasons for low acceptance of routine childhood vaccines during the pandemic are provided in Fig. 1.

**Table 2:** Outcome and predictor variable descriptive statistics for a sample of Canadian parents of children aged ≤ 17 years (N = 2531), October/November 2021.

**Variable**	**Category**	**Total** **n (%)**
**Childhood routine and influenza vaccination intent and receipt**		
Routine childhood vaccination intent/receipt during the COVID-19 pandemic^1^	Intent to receive/receipt	2224 (87.9)
	No intent to receive/no receipt	206 (8.1)
	I don’t know	101 (4.0)
Influenza vaccination intent for child(ren) for the 2021/22 flu season	Agree, more likely to vaccinate	1022 (40.4)
	Undecided	458 (18.1)
	Disagree, less likely to vaccinate	942 (37.2)
	Not eligible	109 (4.3)
Have you experienced discrimination and/or racism when accessing health services for yourself or your child(ren)?	Yes	251 (9.9)
	No	2230 (88.1)
	Don’t know/prefer not to answer	50 (2.0)
**Parents’ perceptions of routine vaccination**		
Has the pandemic changed the way you think about routine vaccines for your child(ren)?^1^	The pandemic has made me realize that routine vaccines are more important	535 (21.1)
	The pandemic has not changed how I think about routine vaccines	1944 (77.0)
	The pandemic has made me realize that routine vaccines are less important	47 (1.9)
I am completely confident that routine vaccines are safe^2^	Agree, more likely to vaccinate	1980 (78.2)
	Neutral	336 (13.3)
	Disagree, less likely to vaccinate	215 (8.5)
Routine vaccination is necessary because vaccine-preventable diseases are common^2^	Agree, more likely to vaccinate	1820 (71.9)
	Neutral	391 (15.4)
	Disagree, less likely to vaccinate	321 (12.6)
Vaccination is a collective action to prevent the spread of disease^2^	Agree, more likely to vaccinate	2167 (85.6)
	Neutral	207 (8.2)
	Disagree, less likely to vaccinate	157 (6.2)
When I think about getting vaccinated, I weigh the benefits and risks to make the best decision possible^2^	Agree, more likely to vaccinate	1995 (78.8)
	Neutral	329 (13.0)
	Disagree, less likely to vaccinate	207 (8.2)
Everyday stress (such as competing priorities or many demands on my time) prevents me from getting vaccinated^2^	Disagree, more likely to vaccinate	1959 (77.4)
	Neutral	311 (12.3)
	Agree, less likely to vaccinate	261 (10.3)
Vaccines are effective^2^	Agree	2122 (83.8)
	Neutral	267 (10.5)
	Disagree	142 (5.6)
It should be mandatory for children to get the recommended childhood vaccines	Agree	1713 (67.7)
	Neutral	416 (16.4)
	Disagree	402 (15.9)
COVID-19 vaccination in Canada should be:	Mandatory for everyone (with exceptions based on medical reasons)	1313 (51.9)
	Mandatory for certain groups (e.g., health care workers)	312 (12.3)
	Mandatory for certain activities (e.g., travel, recreational/social activities)	165 (6.5)
	Voluntary for everyone	669 (26.4)
	I don’t know	72 (2.8)
Have any of your children had COVID-19 disease?	Yes^3^	278 (11.0)
	No	2190 (86.5)
	Don’t know/prefer not to answer	63 (2.5)

Notes.

1 Outcome variable for regression analysis.

2 Parents responded in reference to routine vaccines in general, not specific to childhood routine vaccines.

3 Responses include COVID-19 cases confirmed by COVID-19 testing and presumed positive cases.

### 3.3 Ethnocultural identities and vaccination-related factors

Respondents who self-identified as a Racialized minority or those with two intersecting identities reported the lowest routine childhood vaccination acceptance (16.0 % and 16.1 % respectively). Statistically significant findings included 36.6 % of Indigenous parents who reported that the pandemic made them realize that routine vaccines were more important, compared to 16.7 % of newcomers and 16.9 % of the reference group. Discrimination/racism when accessing health services was most often experienced by Indigenous (27.8 %) and Racialized minority (20.2 %) parents, compared to 4.8 % of the reference group (Table 3).

**Table 3:** Vaccination-related descriptive statistics among ethnocultural identities of Canadian parents of children aged ≤ 17 years (N = 2531), October/November 2021.

**Variable**		**Indigenous** (N = 194) % (CI), n	**Racialized** **minority** (N = 119) % (CI), n	**Newcomer** (N = 138) % (CI), n	**Two** **Intersecting** **identities**^1^ (N = 273) % (CI), n	**Three** **Intersecting** **identities**^2^ (N = 191) % (CI), n	**Reference group**^3^ (N = 1616) % (CI), n
**Routine vaccination acceptance during the pandemic**	Yes	85.1 (79.5–89.5), 165	84.0 (76.7–89.8), 100	85.5 (78.9–90.6), 118	83.9 (79.2–87.9), 229	90.1 (85.2–93.7), 172	89.1 (87.5–90.6), 1440
	No/ uncertain	14.9 (10.5–20.5), 29	16.0 (10.2–23.3), 19	14.5 (9.4–21.1), 20	16.1 (12.1–20.8), 44	9.9 (6.3–14.8), 19	10.9 (9.4–12.5), 176
**How the pandemic has changed the way parents think about routine vaccines for their children**	More important	36.6 (28.6–46.2), 71	24.4 (16.3–35.0), 29	16.7 (10.6–25.0), 23	30.0 (24.2–37.7), 83	29.8 (22.6–38.7), 57	16.9 (14.9–19.0), 273
	No change	62.4 (51.8–74.5) 121	73.9 (59.3–91.1), 88	81.9 (67.5–98.4), 113	67.8 (58.4–78.3), 185	68.6 (57.3–81.4), 131	81.1 (76.8–85.6), 1311
	Less important	1.0 (0.1–3.7) 2	1.7 (0.2–6.1), 2	1.4 (0.2–5.2), 2	2.2 (0.8–4.8), 6	1.6 (0.3–4.6), 3	2.0 (1.4–2.8), 32
**Experiences of discrimination and/or racism when accessing health services**	Yes	27.8 (21.9–34.4), 54	20.2 (13.7–28.0), 24	8.7 (4.8–14.3), 12	19.8 (15.4–24.8), 54	15.7 (11.1–21.4), 30	4.8 (3.8–5.9), 77
	No/ prefer not to answer	72.2 (65.6–78.1), 140	79.8 (72.0–86.3), 95	91.3 (85.7–95.2), 126	80.2 (75.2–84.6), 219	84.3 (78.6–88.9), 161	95.2 (94.1–96.2), 1539

Note. CI = confidence interval

1 Intersecting identities include two of either newcomer, Racialized, or language minority characteristics.

2 Intersecting identities include newcomer, Racialized, and language minority characteristics.

3 Reference group of parents who do not self-identify with these ethnocultural identities (i.e., Indigenous, Racialized minority, newcomer and language minority).

### 3.4 Multivariable logistic regression

Parents who self-identified as a Racialized minority were more than twice as likely to report low routine vaccination acceptance for their children, compared to the reference group (aOR = 2.19, 95 % CI: 1.18–4.05)(Table 4). Younger parents were less likely to experience low routine vaccination acceptance (i.e., they had higher acceptance) compared to parents ≥ 50 years (15–29 years: aOR = 0.35, 95 % CI: 0.19–0.66 and 30–39 years: aOR = 0.59, 95 % CI: 0.37–0.94). Parents who only had preschool-aged children (0–6 years) were less likely to experience low routine vaccination acceptance compared to parents of only school-aged children (7–17 years) (aOR = 0.53, 95 % CI: 0.36, 0.79). No other socioeconomic characteristics were associated with parents’ low routine childhood vaccination acceptance.

**Table 4:** **Unadjusted and adjusted odds ratios for the association between predictor variables and Canadian parents’ routine childhood vaccination acceptance during the pandemic** (low acceptance versus the reference category of high acceptance) (N = 2351).

**Predictor variables**	**Category**	**Unadjusted ORs (95 % CI)**	**Adjusted ORs (95 % CI)**	**p-value**
**Ethnocultural identities** (ref: Reference group)^1^	Racialized minority	1.55 (0.93, 2.60)	**2.19 (1.18, 4.05)**	**0.01**
	Newcomer	1.39 (0.84, 2.28)	1.27 (0.71, 2.27)	0.42
	2 Intersecting identities^2^	**1.57 (1.10, 2.25)**	1.52 (0.96, 2.40)	0.08
	3 Intersecting identities^3^	0.90 (0.55, 1.49)	1.04 (0.56, 1.93)	0.90
	Indigenous	1.44 (0.94, 2.20)	1.41 (0.82, 2.45)	0.22
**Province** (ref: Ontario)	British Columbia	0.66 (0.41, 1.07)	0.61 (0.35, 1.06)	0.08
	Alberta	0.91 (0.60, 1.38)	1.11 (0.68, 1.82)	0.67
	Prairies^4^	1.10 (0.67, 1.81)	0.86 (0.46, 1.61)	0.64
	Quebec	1.01 (0.76, 1.35)	0.95 (0.66, 1.38)	0.80
	Atlantic provinces^5^	1.15 (0.70, 1.89)	1.58 (0.85, 2.93)	0.15
**Age** (ref: ≥ 50 years)	15–29 years	0.70 (0.44, 1.11)	**0.35 (0.19, 0.66)**	**0.001**
	30–39 years	**0.61 (0.43, 0.85)**	**0.59 (0.37, 0.94)**	**0.03**
	40–49 years	0.81 (0.58, 1.13)	0.76 (0.51, 1.15)	0.19
**Gender** (ref: Woman)	Man	1.26 (0.99, 1.60)	1.14 (0.85, 1.53)	0.39
**Marital status** (ref: Married/common-law)	Not married/ common-law	**1.80 (1.35, 2.39)**	1.21 (0.83, 1.75)	0.33
**Household income** (ref: ≥$80,000)	<$40,000	**2.24 (1.58, 3.19)**	1.39 (0.86, 2.23)	0.18
	$40,000- $79,999	**1.63 (1.23, 2.16)**	1.17 (0.82, 1.67)	0.39
	Prefer not to answer	**1.81 (1.17, 2.79)**	1.42 (0.85, 2.37)	0.18
**Highest level of education** (ref: University bachelor's or more)	High school or less	**1.68 (1.19, 2.38)**	0.91 (0.58, 1.43)	0.67
	Non-university certificate/diploma	1.22 (0.93, 1.59)	0.91 (0.65, 1.27)	0.58
**Age category of child(ren)** (ref: School-aged child(ren) 7–17 years)	Preschool-aged child(ren) (0-6y)	**0.51 (0.38, 0.67)**	**0.53 (0.36, 0.79)**	**0.002**
	Pre- & school-aged children (0-17y)	0.82 (0.59, 1.15)	0.94 (0.61, 1.43)	0.76
**Experiences of discrimination and/or racism** (ref: No)	Yes	1.28 (0.88, 1.86)	0.68 (0.43, 1.08)	0.11
	Don’t know/Prefer not to answer	**2.69 (1.41, 5.12)**	1.33 (0.61, 2.91)	0.48
**How the pandemic changed parents’ perceptions about routine childhood vaccines** (ref: Not changed)	Less important	**12.60 (6.88, 23.06)**	**4.16 (1.98, 8.73)**	**<0.0001**
	More important	0.93 (0.69, 1.27)	1.36 (0.92, 1.99)	0.12
**Influenza vaccination intention (**fall 2021/ winter 2022) (ref: Agree)	Disagree	**5.88 (4.12, 8.39)**	**3.46 (2.24, 5.32)**	**<0.0001**
	Neutral	**4.58 (3.06, 6.86)**	**3.42 (2.18, 5.38)**	**<0.0001**
	Not eligible	**3.32 (1.72, 6.43)**	**3.03 (1.39, 6.61)**	**0.005**
**Routine vaccines are effective** (ref: Agree)	Disagree	**7.04 (4.83, 10.26)**	1.03 (0.53, 2.00)	0.93
	Neutral	**6.62 (4.91, 8.92)**	1.18 (0.74, 1.89)	0.49
**I am completely confident that routine vaccines are safe** (ref: Agree)	Disagree	**9.38 (6.77, 13.00)**	**2.95 (1.75, 4.98)**	**<0.0001**
	Neutral	**5.49 (4.08, 7.40)**	1.52 (0.98, 2.36)	0.06
**Routine vaccination is necessary because vaccine-preventable diseases are common** (ref: Agree)	Disagree	**4.53 (3.30, 6.20)**	**2.22 (1.48, 3.31)**	**<0.0001**
	Neutral	**5.36 (4.02, 7.15)**	**1.90 (1.29, 2.80)**	**0.001**
**Vaccination is a collective action to prevent the spread of disease** (ref: Agree)	Disagree	**6.65 (4.64, 9.54)**	1.34 (0.74, 2.42)	0.34
	Neutral	**7.20 (5.23, 9.93)**	1.49 (0.91, 2.42)	0.11
**When I think about getting vaccinated, I weigh benefits and risks to make the best decision possible** (ref: Agree)	Disagree	1.32 (0.87, 2.00)	0.92 (0.52, 1.61)	0.77
	Neutral	**1.77 (1.29, 2.43)**	1.12 (0.73, 1.72)	0.61
**Everyday stress (such as competing priorities or many demands on my time) will prevent me from getting vaccinated** (ref: Disagree)	Agree	**2.54 (1.80, 3.58)**	**2.26 (1.45, 3.52)**	**<0.0001**
	Neutral	**3.93 (2.93, 5.28)**	**2.13 (1.44, 3.16)**	**<0.0001**
**It should be mandatory for children to get the recommended childhood routine vaccines** (ref: Agree)	Disagree	**6.29 (4.72, 8.38)**	**1.85 (1.18, 2.91)**	**0.008**
	Neutral	**3.12 (2.28, 4.29)**	1.07 (0.70, 1.63)	0.76
**COVID-19 vaccination in Canada should be:** (ref: Mandatory for everyone)	Mandatory for certain groups	**1.94 (1.29, 2.92)**	0.98 (0.61, 1.57)	0.93
	Mandatory for certain activities	**2.46 (1.51, 4.00)**	1.60 (0.89, 2.88)	0.11
	Voluntary for everyone	**3.89 (2.92, 5.19)**	1.06 (0.70, 1.60)	0.80
	Don’t know	**5.18 (2.93, 9.14)**	**2.27 (1.12, 4.60)**	**0.02**
**COVID-19 disease status of child(ren)** (ref: No)	Yes	**1.72 (1.23, 2.41)**	1.28 (0.85, 1.95)	0.24
	Prefer not to answer/don’t know	**2.74 (1.53, 4.91)**	1.60 (0.78, 3.26)	0.20

Notes. CI = confidence interval; OR = odds ratio; bold results significant when p ≤ 0.05.

1 Reference group of parents who do not self-identify with any ethnocultural characteristics (i.e., Indigenous, Racialized minority, newcomer and language minority).

2 Intersecting identities include two of either newcomer, Racialized, or language minority.

3 Intersecting identities include newcomer, Racialized, and language minority.

4 Prairie provinces include Saskatchewan and Manitoba.

5 Atlantic provinces include PEI, Nova Scotia, New Brunswick, and Newfoundland and Labrador.

Parents who perceived that routine childhood vaccines were less important because of the COVID-19 pandemic were 4 times more likely to have low vaccination acceptance (aOR = 4.16, 95 % CI: 1.98–8.73). Those who were neutral or did not intend to vaccinate their child against influenza during the pandemic were over three times more likely to experience low vaccination acceptance compared with parents who intended to vaccinate their child against influenza (aOR = 3.42, 95 % CI: 2.18–5.38 and aOR = 3.46, 95 % CI: 2.24–5.32, respectively). Low acceptance of routine vaccines was associated with parents’ perceptions that vaccination was unnecessary (aOR = 2.22, 95 % CI: 1.48–3.31) or unsafe (aOR = 2.95, 95 % CI: 1.75–4.98), and that everyday stress (such as competing priorities or many demands on my time) would prevent vaccination (aOR = 2.26, 95 % CI: 1.45–3.52).

## 4 Discussion

### 4.1 Intersectional characteristics

It is challenging to assess interdependent intersections of social determinants (i.e., ethnocultural identities and social locations), and their associations with routine vaccine acceptance (Rice et al., 2019 Dec, Lane, 2020 Dec). Outside of assessing sociodemographic characteristics (e.g., income, education), compounding social intersections and how these may influence parents’ decision-making and access to routine childhood vaccines have not been readily explored (Carpiano and Bettinger, 2016 Dec, Bell et al., 2020). In the context of the COVID-19 pandemic, parents may have experienced further challenges accessing routine vaccines (MacDonald et al., 2022). Therefore, we intentionally sought to assess intersections of ethnocultural identities and how these may have influenced acceptance of routine childhood vaccination during the pandemic.

### 4.2 Racialized minority and intersectional identities

Parents who self-identified as a Racialized minority, and those with two intersecting identities (i.e., newcomer, language minority, or Racialized minority) reported the lowest acceptance of routine childhood vaccination during the COVID-19 pandemic. We found that Racialized minority parents were twice as likely to report low acceptance, compared to parents who did not report any of the assessed ethnocultural identities. Similarly, Bell et al. (Bell et al., 2020) noted that Racialized minority parents in England experienced increased barriers to accessing routine vaccines during the pandemic, subsequently influencing their low acceptance. A study in the United States (He et al., 2022 Feb) also noted increased routine childhood vaccine hesitancy and risk perceptions among Racialized minority parents and those with lower household incomes. We found no association between parents’ social locations (i.e., income, education) and acceptance of routine childhood vaccination. However, younger parents in our study (15–39 years), and those with only preschool-aged children (0–6 years), reported higher acceptance compared to parents ≥ 50 years and those who only had school-aged children (7–17 years). Younger children, compared to older children, receive a higher number of recommended routine vaccines, and have an increased risk of morbidity and mortality from vaccine-preventable diseases. Therefore, parents in our study with young children may be more motivated to accept vaccination. Conversely, research has shown increased hesitancy and incomplete childhood vaccination among younger parents (Khan et al., 2017, Funk, 2017). Further research regarding the association of parents’ age and routine vaccine acceptance is required.

### 4.3 Indigenous parents

Low routine childhood vaccination coverage levels have been shown within some Indigenous populations in Canada (Rudnick et al., 2022 Jan 31, Carpiano and Bettinger, 2016 Dec). However, data is limited and health services have a long history of discriminatory practices directed at Indigenous peoples, causing significant harm and mistrust for some First Nations, Métis, and Inuit peoples (Allan and Smylie, 2015, Adelson, 2005 Mar, Nunn, 2018 Nov). Indigenous parents in our study had high intentions to vaccinate their children, and were significantly more likely to report that the pandemic has made them realize that routine vaccines were more important, compared to the reference group of parents. A Canadian study (MacDonald et al., 2022) that assessed routine childhood vaccination processes in a First Nations community noted that parents were highly motivated and working hard to vaccinate their children. However, this study showed that entrenched colonial processes and policies placed unrealistic expectations on families, in order to receive childhood vaccines. For example, prior to the pandemic, “one child, one appointment” policies were a noteworthy barrier to accessing childhood vaccination for some First Nations parents (MacDonald et al., 2022). This challenge may have been exacerbated during the pandemic when “one parent, one child, one appointment” policies further inhibited siblings and/or additional parents from attending vaccination appointments or utilizing waiting room areas (Bell et al., 2020). How impossible it might seem, then, for highly motivated parents to access childhood vaccines considering school and daycare closures that might otherwise support “one parent, one child” vaccination appointments? The COVID-19 pandemic serves as an opportunity to highlight the necessity of inclusive and accessible vaccination services for families.

### 4.4 Discrimination when accessing health services

In Canada over 25 % of the population have reported experiences of discrimination based on ethnocultural identities and social locations; racial discrimination being most commonly reported (Government of Canada. Public Health Agency of Canada., 2021, Hyman et al., 2019). Parents in our study who self-identified as Indigenous or a Racialized minority experienced discrimination/racism significantly more often when accessing health services, compared to the reference group. Similarly, when assessing COVID-19 vaccination intentions in Canada, Lin (Lin, 2022 Aug) found a two-fold increase in newcomers' perceptions of anticipating racial stigmatization. Another Canadian study found that many Racialized minorities and newcomers experienced discrimination, which subsequently negatively influenced their health-related psychological, social, and environmental quality of life (Zghal et al., 2021 Sep). Importantly, an association exists between past experiences of discrimination and decreased health service seeking behaviors (Kholina et al., 2022 Dec, Williams et al., 2019 Dec).

The 2019 Chief Public Health Officer’s Report on the State of Public Health in Canada (Government of Canada. Public Health Agency of Canada., 2021) noted that although public health policies and programs should benefit all persons, health inequalities exist within some ethnocultural populations, often as a result of health systems and social discriminations that hinder access (Ismail et al., 2020). These inequities were highlighted during the pandemic, with regards to accessing COVID-19 related health services (Kholina et al., 2022 Dec). For example, a study from Israel (Luxenburg et al., 2023 Jan) found decreased COVID-19 testing, vaccination, and an increase in confirmed COVID-19 disease among Racialized minorities and those with a lower socioeconomic status. This study demonstrated how intersections of low socioeconomic status and Racialized identities were associated with widening health disparities during the pandemic. Similarly, a Canadian study identified how intersecting forms of discrimination constrained COVID-19 vaccination decision-making for Racialized minority and Indigenous peoples (Manca et al., 2022).

Before the COVID-19 pandemic, barriers to routine childhood vaccination for some ethnocultural groups were identified as decreased access to health services (i.e., language barriers, inadequate transportation), vaccine hesitancy, gaps in vaccination service knowledge, and other cultural determinants (i.e., religious beliefs) (Pandey et al., 2022 Feb, Wilson et al., 2018). Therefore, culturally relevant services are required to support meaningful engagement in vaccination decision-making (Kholina et al., 2022 Dec, Wilson et al., 2018).

### 4.5 Routine vaccination acceptance among all parents

Routine childhood vaccination acceptance during the COVID-19 pandemic (Oct/Nov 2021) among our full sample of parents (N = 2531) was higher than prepandemic 7-year-old children and 14-year-old adolescents national coverage levels (Government of Canada. Public Health Agency of Canada., 2022). Although vaccination intention does not necessarily translate to uptake, it is noteworthy that almost a quarter of parents in our study reported that the COVID-19 pandemic made them realize that routine vaccines were more important. A study from the United Kingdom also reported increases in parents’ acceptance of routine vaccines for their children during the pandemic (McQuaid et al., 2022). Increasing acceptance is attributed to growing awareness of the importance and effectiveness of childhood vaccines due to the spotlight on COVID-19 vaccination and its role in preventing adverse disease outcomes and enabling a return to normal socioeconomic activities (Abu-rish et al., 2022, McQuaid et al.,). Our study’s finding of parents’ increasing acceptance is critical in light of the decrease in routine childhood vaccine uptake that occurred early in the COVID-19 pandemic (Abu-rish et al., 2022, McDonald et al., 2020, Kiely et al., 2022). Importantly, many countries who reported the alarming decrease, had returned to prepandemic levels, or higher, by January 2021 (Kiely et al., 2022, MacDonald et al., 2022). This rebound may be due to effective public health communication, improved access to vaccines, and parents who were increasingly motivated to vaccinate their children (Harrison and Wu, 2020 Apr, Bell et al., 2020, Zhang et al.,).

### 4.6 Reasons for low acceptance among all parents

Of the overall sample of parents, 12.1 % reported low acceptance of routine childhood vaccination during the pandemic. A small percentage of these reported that either they do not vaccinate their children at all or lack confidence in the safety or necessity of vaccines. Most parents cited COVID-19 related disruptions (i.e., health centre closures) or uncertainties (i.e., COVID-19 transmission at the appointment) as their rationale for low acceptance. Our findings are similar to other studies (Lee et al., 2022, Bell et al., 2020), that found parents’ acceptance of routine childhood vaccines was negatively impacted by concerns of COVID-19 transmission during vaccination appointments, and a lack of clarity around the availability of services during times of restrictive public health measures. Furthermore, parents’ low acceptance was significantly associated with beliefs that routine vaccination was unnecessary, unsafe, and that everyday stress prevented access. Our results are similar to research that shows parents’ hesitancy is often rooted in concerns about the safety and efficacy of vaccines, rather than an outright refusal (Kempe et al., 2020). Parents' concerns regarding accessing routine vaccines may also reflect the timing of our survey (Oct/Nov 2021), when the swiftly circulating delta variant (B.1.617.1) resulted in further public health restrictive measures aimed to protect against a new wave of COVID-19 disease.

Parents’ low routine vaccination acceptance was associated with low seasonal influenza vaccination acceptance. Parents were equally likely to accept or decline influenza vaccination for their child during the pandemic, with a large majority who remained undecided. Similarly, a study from the United States (Sokol and Grummon, 2020 Dec 1) found that the COVID-19 pandemic may amplify polarity in childhood influenza vaccine acceptance. For instance, parents may prioritize routine and COVID-19 vaccines over concerns of multiple vaccinations, thereby delaying influenza vaccination (He et al., 2022 Feb, Santibanez et al., 2020). Others may prioritize influenza vaccination for their child, seeking protection against a potential second respiratory illness during the pandemic (Goldberg et al., 2022, Wang et al., 2022 Sep 2).

In the context of the COVID-19 pandemic, it is important to understand how routine childhood vaccine hesitancy may have been perpetuated. Parents in our study with low acceptance were almost four times as likely to report that they believed routine childhood vaccination was less important because of the pandemic. Similarly, a study from the UK found that many parents believed their children were at less risk for acquiring vaccine-preventable diseases due to social distancing measures (Bell et al., 2020). Furthermore, mandated COVID-19 vaccination and measures implemented to prevent the spread of COVID-19 disease created significant social and economic hardship for many parents (He et al., 2022 Feb). Although mandates and public health measures aimed to protect the greater population, some parents may have been disproportionately impacted, subsequently influencing decision-making and beliefs about vaccination.

### 4.7 Implications

Early COVID-19 vaccination in Canada was based on equitable allocation to populations who may have had differential access to health services, such as remote communities, Indigenous populations, and other identified at-risk groups (Government of Canada, 2021, Kholina et al., 2022 Dec). This vaccination prioritization framework aimed to reduce health inequities and prevent further discrimination within some populations (Government of Canada, 2021). Similarly, public health officials in Canada should consider a routine childhood vaccination framework that prioritizes equitable access for some ethnocultural groups, where known social discriminations and inequities to accessing services exist.

### 4.8 Strengths and limitations

We collected novel information from a nationally representative sample of parents Oct/Nov 2021 during the COVID-19 pandemic, just before the first pediatric COVID-19 vaccine was approved for children aged 5–11 years in Canada. Minimum recruitment quotas ensured adequate representation of parent groups in our study (Indigenous, Racialized and language minorities, and newcomers). Our study captured how parents’ perceptions and acceptance of routine childhood vaccines were influenced by the COVID-19 pandemic, including experiences of discrimination/racism when accessing health services, and how these factors differed across ethnocultural groups. Our sample was selected from a pre-existing panel that excludes respondents who do not have access to internet or strong reading proficiency in English or French. Therefore, results may not reflect those of the larger Canadian population, nor those who may experience socioeconomic inequities relevant to ethnocultural groups included in this study. Data were self-reported, therefore some variables may be affected by recall and desirability bias. Parents’ acceptance of routine vaccines for their children (i.e., intentions and receipt of vaccines) were collected as one variable in our survey. Intentions to vaccinate may not necessarily result in future receipt of vaccines, therefore further research is required to better understand whether differences in parents’ perceptions and intentions for their children influence the behavior of vaccine uptake.

## 5 Conclusions

Before the COVID-19 pandemic, some ethnocultural groups in Canada experienced low acceptance and access to routine childhood vaccination, where one's social location and associated discriminations may have contributed to inequities in uptake. The pandemic may have further exacerbated these challenges for some parents. Under the spotlight of the pandemic, public health decision-makers should ensure equitable access to routine childhood vaccination that targets the inclusion of ethnocultural groups, who may experience disproportionate barriers to services.

## 6 Authors’ statement

All authors attest that they meet the ICMJE criteria for authorship.

## Funding

This work was funded by the Canadian Institutes of Health Research [grant number 172700].

## Declaration of Competing Interest

The authors declare that they have no known competing financial interests or personal relationships that could have appeared to influence the work reported in this paper.

## Data Availability

The authors do not have permission to share data.
