# Supplementary material for: Routine childhood vaccination among ethnocultural groups in Canada during the COVID-19 pandemic: A national cross-sectional study
Source: Prev Med Rep. 2023 Sep 25;36:102435. doi: 10.1016/j.pmedr.2023.102435 (PMC10562748; doi:10.1016/j.pmedr.2023.102435)
Supplement: Supplementary data 1 [file mmc1.docx]

**Supplementary material**

**Table A1. The Checklist for Reporting Results of Internet E-Surveys (CHERRIES)**(27)

| **Item** | **Explanation** | **Response** |
| --- | --- | --- |
| **Designs** | | |
| Describe survey design | Describe target population, sample frame. Is the sample a convenience sample? (In “open” surveys this is most likely.) | Respondents were randomly selected from the Leger Web panel, which has more than 200,000 panelists in Quebec and more than 400,000 panelists across Canada. The target population included parents of children younger than 7 years old (N=750); parents of children 7-17 years old (N=750); people < 60 years, not pregnant and with no chronic medical conditions; Adults > 60 years old (N=500); healthcare workers (N=500); Indigenous persons (N=300); Pregnant respondents (N=200); racialized minority persons (N=500) (persons, other than Indigenous peoples, who are non-Caucasian in race or non-white in colour); newcomers (N=500) (arrived in Canada within the past 5 years); persons with chronic medical conditions, age <60 years (N=500); persons with disabilities (N=500); and persons whose first language is not English or French (N=500) |
| **Institutional Review Board approval and informed consent process** | | |
| IRB approval | Mention whether the study has been approved by an IRB. | Health Research Ethics Board, University of Alberta |
| Informed consent | Describe the informed consent process. Where were the participants told the length of time of the survey, which data were stored and where and for how long, who the investigator was, and the purpose of the study? | Participants were informed responses are confidential and stored in the Leger Opinion data base by unique number, length of survey (15-20 minutes), and information was provided regarding the purpose of the study and principal investigators. Data provided to the COVImm project is confidential, anonymous, and contains no identification information unless participant contact information provided in response to a survey question. |
| Data protection | If any personal information was collected or stored, describe what mechanisms were used to protect unauthorized access. | Data received from Leger is stored in the University of Alberta, Faculty of Nursing, [Health Research Data Repository (HRDR](https://www.ualberta.ca/nursing/research/supports-and-services/hrdr.html#:~:text=Based%20within%20the%20Faculty%20of,health%20research%20data%20and%20meta%2D)) which is a secure and confidential virtual research environment. Researchers can securely access data, however information cannot be removed or copied from HRDR unless by request and permission granted |
| **Development and pre-testing** | | |
| Development and testing | State how the survey was developed, including whether the usability and technical functionality of the electronic questionnaire had been tested before fielding the questionnaire. | The survey was based on a previous national survey of Canadians’ acceptance of routine childhood vaccines and a literature review of validated conceptual models and measures of vaccine up-take behaviours. The 5Cs(28) psychological antecedents of vaccination model was chosen as the most comprehensive validated measure of vaccine behaviours. The 5Cs model is grounded in vaccine acceptance and hesitancy theoretical models and includes 5 category measures of vaccine uptake including confidence, complacency, constraints, calculation, and collective responsibility. The 5Cs model has been tested internationally in 4 different studies and includes a long (15-item) and short (5-item) scale. Cronbach’s α was used as an indicator of reliability of the 5Cs scale, and correlation analysis for assessing the concurrent and construct validity as well as for the comparison to existing measures of vaccination behaviours. The survey was pre-tested, then validated on 20 members of the public, and revised as needed. Non-discriminatory and inclusive language was used throughout the survey vetted by Dr. Devon Greyson an expert in health and social equity. |
| **Recruitment process and description of the sample having access to the questionnaire** | | |
| Open survey versus closed survey | An “open survey” is a survey open for each visitor of a site, while a closed survey is only open to a sample which the investigator knows. | Closed survey: Respondents (N=6026) were randomly selected from the Leger Opinion panel which consists of 400,000 members from every province and territory of Canada. |
| Contact mode | Indicate whether or not the initial contact with the potential participants was made on the Internet. | An invitation email in English or French was sent to panelists with a custom web link to access the survey. The link remained active for a given period of time and included a unique identification number for each respondent. Respondents completed the survey at their leisure, in one sitting. |
| Advertising the survey | How/where was the survey announced or advertised? | No external advertising outside of the existing Leger Opinion Panel. |
| **Survey administration** | | |
| Web/E-mail | State the type of e-survey (eg, posted on a Web site/sent through e-mail). If it is an e-mail survey, were the responses entered manually into a database, or was there an automatic method for capturing responses? | The email invitation contains a customized weblink to access the survey. The link is active for a given period of time and is unique to each respondent (it includes an ID number). Answers are automatically recorded, and respondents can answer the questionnaire at their convenience. |
| Context | Describe the Web site in which the survey was posted. | Not applicable |
| Mandatory/ voluntary | Was it a mandatory survey to be filled in by every visitor who wanted to enter the Web site, or was it a voluntary survey? | A randomly selected and voluntary survey. |
| Incentives | Were any incentives offered (eg, monetary, prizes, or non-monetary incentives such as an offer to provide the survey results)? | Leger Opinion Panel members earn money, AirMiles or Aeroplan points for each survey completed, and are eligible to win one of 5 monthly prizes valued at $2,500 (Opinion Panel [website](https://leger360.com/services/legeropinion-leo/)) |
| Time/Date | In what timeframe were the data collected? | October 14^th^ - November 12^th^, 2021 |
| Randomization of items or questionnaires | To prevent biases items can be randomized or alternated. | The questionnaire was administered in a meaningful order; items were not randomized or alternated. |
| Adaptive questioning | Use adaptive questioning (certain items, or only conditionally displayed based on responses to other items) to reduce number and complexity of the questions | Branching questions were conditionally displayed based upon responses to other items, and specific questions administered to target populations only. |
| Number of items | What was the number of questionnaire items per page? The number of items is an important factor for the completion rate. | One question item per page. |
| Number of screes (pages) | Over how many pages was the questionnaire distributed? The number of items is an important factor for the completion rate. | Approximatley 75 pages for 75 questions (including 2 open-ended questions). |
| Completeness check | It is technically possible to do consistency or completeness checks before the questionnaire is submitted. Was this done, and if “yes”, how? An alternative is to check for completeness after the questionnaire has been submitted. If this has been done, it should be reported. All items should provide a non-response option such as “not applicable” or “rather not say”, and selection of one response option should be enforced. | Respondents could only move forward in the questionnaire once an answer was provided for the displayed question. Incomplete surveys were discarded and not included in data collection. Batter questions monitored for and eliminated ‘straight liners.” Survey participants were monitored for inattentiveness and data excluded for respondents who gave no substantive comments to open-ended questions. The survey had to be completed in on sitting. Although no lime limits were required for this study, Leger kept track of how long respondents take to complete the questionnaire. |
| Review step | State whether respondents were able to review and change their answers. | Respondents could only move forward in the questionnaire once an answer was provided for the displayed question. |
| **Response rates** | | |
| Unique site visitor | If you provide view rates or participation rates, you need to define how you determined a unique visitor. There are different techniques available, based on IP addresses or cookies or both. | Each respondent had a unique URL. Incomplete surveys were discarded and not included in data collection. |
| View rate  (Ratio of unique survey visitors/unique site visitors) | Requires counting unique visitors to the first page of the survey, divided by the number of unique site visitors. | Not applicable. |
| Participation rate | Count the unique number of people who filled in the first survey page, divided by visitors who visit the first page of the survey. This can also be called “recruitment” rate. | Not applicable |
| Completion rate (Ratio of users who finished the survey/users who agreed to participate) | The number of people submitting the last questionnaire page, divided by the number of people who agreed to participate. This is only relevant if there is a separate “informed consent” page or if the survey goes over several pages. This is a measure for attrition. Note that “completion” can involve leaving questionnaire items blank. This is not a measure for how completely questionnaires were filled in. | Incidence rates could not be displayed as a single figure due to mixed population quota groups and the heterogenous nature of the target population. |
| **Preventing multiple entries from the same individual** | | |
| Cookies used | Indicate whether cookies were used to assign a unique user identifier to each client computer. If so, mention the page on which the cookie was set and read, and how long the cookie was valid. Were duplicate entries avoided by preventing users access to the survey twice; or were duplicate database entries having the same user ID eliminated before analysis? | Unique URL’s were required for each respondent. The email invitation contains a customized web link to access the survey. The link is active for a given period of time and is unique to each respondent (it includes an ID number). |
| IP check | Indicate whether the IP address of the client computer was used to identify potential duplicate entries from the same user. If so, mention the period of time for which no two entries from the same IP address were allowed. Were duplicate entries avoided by preventing users with the same IP address access to the survey twice; or were duplicate database entries having the same IP address within a given period of time eliminated before analysis? If the latter, which entries were kept for analysis? | (see above). |
| Log file analysis | Indicate whether other techniques to analyze the log file for identification of multiple entries were used. If so, please describe. | Quality control mechanisms:  • Telephone validation occurred for 15% of respondents to validate their identity and profile.  • Online respondents agreed to phone validation prior to completing the survey in order to be included in completes.  • The sample meet a fail rate of less than 10% (or validation continued until fail rate was less than 10%).  • Those that fail validation were replaced. Validation included confirming identity and re-asking screener questions.  • Embedded consistency questions cross referenced to the panel data base, to ensure the survey was completed by the correct person. |
| Registration | In “closed” surveys, users need to login first and it is easier to prevent duplicate entries from the same user. Describe how this was done. For example, was the survey never displayed a second time once the user had filled it in, or was the username stored together with the survey results and later eliminated? If the latter, which entries were kept for analysis? | The survey was never displayed a second time once the user had completed the questionnaire. |
| **Analysis** | | |
| Handling of incomplete questionnaires | Were only completed questionnaires analyzed? Were questionnaires which terminated early also analyzed? | Only completed questionnaires were included in data collection and analyzed. Questionnaires that were terminated early were discarded. |
| Questionnaires submitted with an atypical timestamp | Some investigators may measure the time people needed to fill in a questionnaire and exclude questionnaires that were submitted too soon. Specify the timeframe that was used as a cut-off point, and describe how this point was determined. | Respondents answered the questionnaire at their own convenience, however the survey had to be completed in one sitting. |
| Statistical correction | Indicate whether any methods such as weighting of items or propensity scores have been used to adjust for the non-representative sample; if so, please describe the methods. | Leger Web’s random sample was weighted according to gender, age, region, mother tongue, education and presence of minor children in the household (or any other sociodemographic element discussed with the research team) including those screened out for answering the survey in order to obtain a sample representative of the entire studied populations [using the latest census data from Statistics Canada]. |

**Table A2. Survey questions**

| **Variable** | **Question and response options** |
| --- | --- |
| Self-identified ethnicity | **What is your ethnic or cultural origin?** (Select all that apply)  White (e.g., Caucasian, European, etc.), Black (e.g., African, Haitian, Jamaican, etc.) Latin / Central American (e.g., Mexican, Colombian, Brazilian, Cuban, etc.), Arabic/West Asian/North African (e.g., Armenian, Egyptian, Iranian, Lebanese, Moroccan, etc.), East Asian (e.g., Chinese, Filipino, Japanese, Korean, Vietnamese, etc.), South Asian (e.g., Indian, Sri Lankan, etc.), other please specify, prefer not to answer |
| Parents new to Canada | **For respondents not born in Canada: When did you come to Canada?**  Between 2016 and 2020; Between 2011 and 2015; Before 2011 |
| Citizenship status | **What is your citizenship status in Canada?**  Canadian citizen, landed immigrant (permanent resident), refugee (asylum seeker), non-permanent or temporary resident (e.g., work or study visa), Other please specify, prefer not to answer |
| Language spoken most often at home | **Which language do you speak most often at home?** (drop-down list, select one)  English, French, Anishinini, Atikamekw, Dakota/Sioux, Dene, Innu, Inuktitut, Mi’kmaq, Michif, Nehiyawewin (Cree), Ojibwe, Siksiká (Blackfoot), Arabic, Cantonese, German, Gujarati, Farsi, Italian, Korean, Mandarin, Polish, Portuguese, Punjabi, Russian, Spanish, Tagalog, Tamil, Urdu, Vietnamese, Other please specify |
| Province | **In which province or territory do you live?**  British Columbia; Alberta; Saskatchewan; Manitoba; Ontario; Quebec; New Brunswick; Nova Scotia; Prince Edward Island; Newfoundland; Northwest Territories; Yukon; Nunavut |
| Age | **What is your age?** (drop-down select age)  Drop down answers for age of respondent |
| Highest level of education | **What is the highest level of education you have completed?**  Some high school or less; High school diploma or equivalent; Registered Apprenticeship or other trades certificate or diploma; College, CEGEP or other non-university trade, certificate, or diploma; University certificate or diploma below bachelor's level; Bachelor's degree; Post graduate degree above bachelor's level; Prefer not to answer |
| Employment status | **Which of the following categories best describes your current employment status?**  Working full-time (35 or more hours per week); Working part-time (less than 35 hours per week); Currently unemployed; Prefer not to answer |
| Annual household income | **To the best of your knowledge, what is the total combined income before tax of everyone living in your household?**  $19,999 or less; Between $20,000 and $39,999; Between $40,000 and $59,999; Between $60,000 and $79,999; Between $80,000 and $99,999; Between $100,000 and $249,000; $250,000 or more; Prefer not to answer |
| Gender | **What is your gender?** (Select all that apply)  Woman, Man, Gender non-conforming, Transgender, Two-spirit, Not listed please specify |
| Marital status | **What is your current marital status?**  Single, Married/Common law, Divorced/Separated, Widowed, Prefer not to answer, Not listed please specify |
| Number of children 0-17 years old in the household | **Are you the parent/primary guardian (e.g., birth parent, foster parent, stepparent, adoptive parent) who makes the health care decisions for one or more children 17 years old or younger?** (If yes, drop down answers for number and age of each child) |
| Parents’ routine vaccination intention for their children during the COVID-19 pandemic | **If your child was due to receive a routine vaccine (e.g., MMR/ measles, whooping cough, rotavirus) during the pandemic (since March 2020) did you, or would you, have them get it?**  Yes, my child was due for a routine vaccine and they received it, or I would have them get it if one was due; No, they did not receive it, or I would not have them get it if one was due; I don’t know |
| Parents’ influenza vaccine intention for their children | **Do you agree or disagree with the following: I received or plan to get the seasonal flu vaccine for my child this year (Fall 2021 or Winter 2022)?**  Strongly disagree, disagree, neither agree nor disagree, agree |
| Parents’ experience of discrimination and/or racism | **Have you experienced discrimination and/or racism when accessing health services for yourself or your child(ren)?**  Yes; No; Don’t know; prefer not to answer |
| The pandemics impact on parents’ perceptions of childhood routine vaccines | **Has the pandemic changed the way you think about routine vaccines for your child?**  The pandemic has not changed how I think about childhood routine vaccines; The pandemic has made me realize that childhood routine vaccines are more important; The pandemic has made me realize that childhood routine vaccines are less important; I don’t know; Other, please specify |
| Confidence in the safety of routine vaccines | **I am completely confident that routine vaccines are safe**  Strongly disagree, disagree, neither agree nor disagree, agree, strongly agree |
| Necessity of routine vaccination | **Routine vaccination is unnecessary because vaccine-preventable diseases are not common anymore**  Strongly disagree, disagree, neither agree nor disagree, agree, strongly agree |
| Vaccination as a collective action | **Vaccination is a collective action to prevent the spread of disease**  Strongly disagree, disagree, neither agree nor disagree, agree, strongly agree |
| Active routine vaccination decision-making | **When I think about getting vaccinated, I weigh the benefits and risks to make the best decision possible**  Strongly disagree, disagree, neither agree nor disagree, agree, strongly agree |
| Constraints preventing access to routine vaccines | **Everyday stress (such as competing priorities or many demands on my time) prevents me from getting vaccinated**  Strongly disagree, disagree, neither agree nor disagree, agree, strongly agree |
| Routine vaccine effectiveness | **Vaccines are effective**  Strongly disagree, disagree, neither agree nor disagree, agree, strongly agree |
| Mandated routine vaccination | **It should be mandatory for children to get the recommended childhood vaccines**  Strongly disagree, disagree, neither agree nor disagree, agree, strongly agree |
| Mandated COVID-19 vaccination | **COVID-19 vaccination in Canada should be:**  Mandatory for everyone (with exceptions based on medical reasons); Mandatory for certain groups (e.g., health care workers); Mandatory for certain activities (e.g., travel, recreational/social activities); Voluntary for everyone; I don’t know |
| COVID-19 disease status | **Have any of your children had COVID-19 disease?**  Yes; No; Don’t know; Prefer not to answer |
| Parents’ reasons for no intention/no receipt of childhood routine vaccines during the pandemic | **What is the main reason you did not/would not get routine vaccines for your child?**  Worried about COVID-19 transmission at the appointment; Not sure if vaccination appointments still happening; Health centre/doctor cancelling my child's vaccination appointment; Waiting until the pandemic was over; Difficulty getting to the appointment (e.g. due to time, transportation, etc); I don’t give my child vaccines; Other, please specify (additional option provided for parents of a child(ren) aged 7-17 years: Schools closed so my child could not get the vaccine) |

**Table A3. Ethnocultural characteristics of parents of children ≤17 years old (N=2531)**

| **Characteristic** | **Category** | **Total n (%)** |
| --- | --- | --- |
| Language spoken most often at home | English | 1487 (58.8) |
|  | French | 790 (31.2) |
|  | Cantonese | 38 (1.5) |
|  | Urdu | 27 (1.1) |
|  | Spanish | 21 (0.8) |
|  | Tagalog | 20 (0.8) |
|  | Mandarin | 19 (0.7) |
|  | Punjabi | 13 (0.5) |
|  | Russian | 13 (0.5) |
|  | Arabic | 12 (0.5) |
|  | Hindi | 12 (0.5) |
|  | Gujarati | 11 (0.4) |
|  | Portuguese | 7 (0.3) |
|  | Tamil | 7 (0.3) |
|  | Indigenous languages^1^ | 6 (0.2) |
|  | Malayalam | 5 (0.2) |
|  | Farsi and/or Dari | 4 (0.2) |
|  | Romanian | 4 (0.2) |
|  | Vietnamese | 4 (0.2) |
|  | German | 3 (0.1) |
|  | Afrikaans | 2 (0.1) |
|  | Bengali | 2 (0.1) |
|  | Berbere | 2 (0.1) |
|  | Estonian | 2 (0.1) |
|  | Italian | 2 (0.1) |
|  | Telugu | 2 (0.1) |
|  | Yoruba | 2 (0.1) |
|  | Other languages^2^ | 14 (0.6) |
| Self-identified ethnicity | European | 1771 (70.0) |
|  | East Asian | 193 (7.6) |
|  | South Asian | 145 (5.7) |
|  | Black | 87 (3.4) |
|  | Latin | 37 (1.5) |
|  | Arabic | 55 (2.1) |
|  | First Nations | 113 (4.5) |
|  | Métis | 75 (3.0) |
|  | Inuk | 4 (0.2) |
|  | Mixed ethnicity | 37 (1.4) |
|  | Prefer not to answer | 14 (0.6) |
| Location of residence for Indigenous parents (n=195) | Reservation, settlement of community | 33 (16.9) |
|  | Urban area | 114 (58.5) |
|  | Rural area | 43 (22.0) |
|  | Prefer not to answer | 5 (2.6) |

^1^ Indigenous languages include Dakota/Sioux, Innu, Anishinini, and Cree

^2^ Other languages include (n=1) each of the following: Albanian, Bulgarian, Edo, Igbo, Indonesian, Japanese, Kannada, Korean, Latvian, Marathi, Polish, Serbian, Turkish, and Ukrainian
